# Severe Ulcerative Colitis Complicated by Malakoplakia Resulting in Proctocolectomy

**DOI:** 10.14309/crj.0000000000001257

**Published:** 2023-01-12

**Authors:** Lotus Alphonsus, Theshani A. De Silva, David Driman, Vipul Jairath

**Affiliations:** 1 Schulich School of Medicine and Dentistry, Western University, London, Ontario, Canada; 2 Pathology and Laboratory Medicine, Schulich School of Medicine and Dentistry, Western University and London Health Sciences Centre, London, Ontario, Canada; 3 Division of Gastroenterology, Department of Medicine, Schulich School of Medicine and Dentistry, Western University, London, Ontario, Canada; 4 Lawson Health Research Institute, Western University, London, Ontario, Canada; 5 Department of Epidemiology and Biostatistics, Western University, London, Ontario, Canada

**Keywords:** ulcerative colitis, malakoplakia

## Abstract

We report a 45-year-old man with medically refractory ulcerative colitis with superimposed colonic malakoplakia, presumed related to chronic use of azathioprine and biologics. This is the first reported case of malakoplakia in a patient requiring high doses of combination therapy. Treatment of malakoplakia is not standardized, but can involve systemic antibiotics, or surgical resection, which in this case resulted in proctocolectomy.

## INTRODUCTION

Malakoplakia is a rare granulomatous inflammatory condition with a poorly understood etiology and pathogenesis. It commonly occurs in the urinary tract and gastrointestinal tract and is believed to be associated with a defect in the bactericidal activity of macrophages with *Escherichia coli* reported as the most common infecting agent.^1^ This case study reports a patient with medically refractory ulcerative colitis (UC) with superimposed colonic malakoplakia, presumed related to chronic use of azathioprine and biologics.^2–4^ Treatment is not standardized, but can involve systemic antibiotics, or surgical resection, as demonstrated with our patient.^1^

## CASE REPORT

A 45-year-old man with pan-UC diagnosed in 2005 received long-term treatment with 5-aminosalicylic acid and azathioprine 150 mg once daily for over 10 years. In 2016, he started vedolizumab while remaining on azathioprine because of disease worsening. He received maintenance therapy with vedolizumab 300 mg every 8 weeks, which was later escalated in 2018 to every 4 weeks without improvement. Repeat endoscopic assessment persistently demonstrated a Mayo endoscopic score of 3. His condition was complicated by 3 episodes of *Clostridium difficile* infection within 18 months in 2019, requiring vancomycin. In June 2020, sigmoidoscopy revealed a new ulcerated, stenotic region in the sigmoid colon, with a Mayo endoscopic score of 3 and biopsies confirming chronic active colitis, without dysplasia or malignancy. Vedolizumab was stopped in May 2020, and he was switched to ustekinumab, initially 90 mg every 8 weeks, which was then escalated because of persistent disease activity to every 4 weeks in September 2020. Response to ustekinumab was assessed in January 2021, with sigmoidoscopy demonstrating continuous colitis from the anal verge to the point of insertion at 60 cm, with a Mayo endoscopic score of 3. Because of ongoing disease activity, in January 2021, he was switched to infliximab and after induction received a maintenance regimen of 5 mg/kg every 8 weeks in combination with azathioprine. Repeat sigmoidoscopy 6 months later demonstrated worsening stenosis in the sigmoid colon, Mayo endoscopic score of 3, with biopsies reporting chronic active colitis and a new finding of malakoplakia. Targeted mucosal biopsies and computed tomography of the abdomen showed no evidence of neoplasia. Because of the presence of malakoplakia, azathioprine was stopped in February 2021, and Infectious Disease was consulted. As long-term antibiotics have been shown in the literature to improve malakoplakia, the patient was started on rifaximin 200 mg twice a day. Although ciprofloxacin has been used with some success, it was considered a risk of recurrent *C. difficile* infection in this patient. Of note, he experienced worsening diarrhea on rifaximin, and it was recommended that he reduce his dose to 200 mg once daily to help with symptoms. He completed his course of rifaximin in October 2021, and since then, the repeat biopsies have not shown any evidence of malakoplakia. Despite the use of 3 biologics, treatment for *Clostridium difficile* and malakoplakia, the patient remained symptomatic and, because of the presence of colonic stenosis and persistent endoscopic inflammation, underwent successful laparoscopic proctocolectomy and permanent end ileostomy in April 2022. There was no evidence of dysplasia or malignancy in the colonic resection specimen. The patient has been doing remarkable well since his end-ileostomy procedure. We recently completed an ileoscopy in October 2023, which showed normal ileal mucosa with no evidence of inflammation or ulceration.

## DISCUSSION

**Figure 1.**
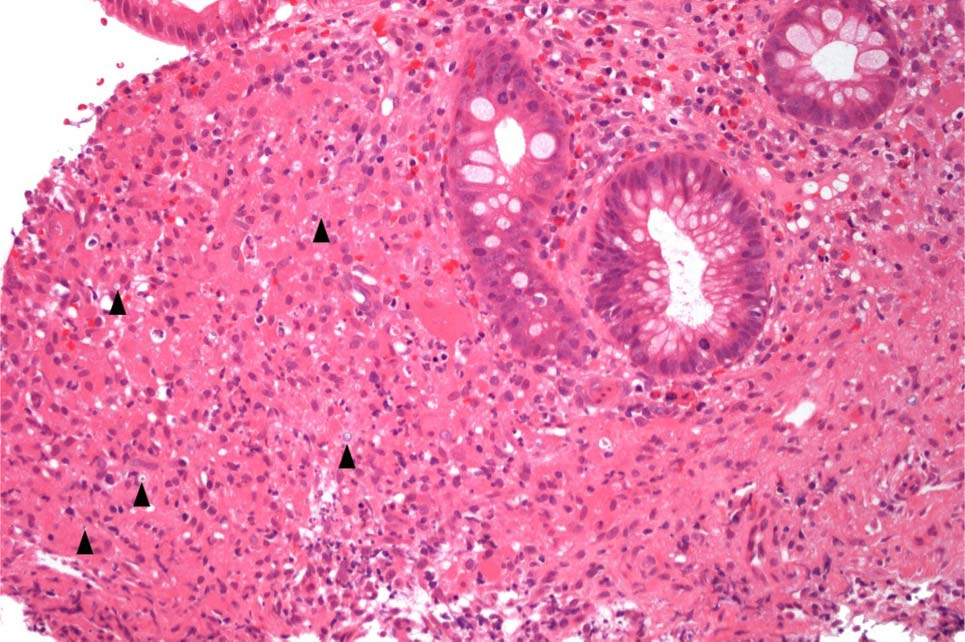
Colonic mucosa with crypt distortion (from ulcerative colitis) and malakoplakia, characterized by diffuse infiltration of the mucosa by eosinophilic histiocytes, some of which contain gray, round calcospherites (Michaelis-Gutmann bodies) (arrowheads), representing undigested bacterial elements associated with calcium and iron deposition. 20× magnification.

**Figure 2.**
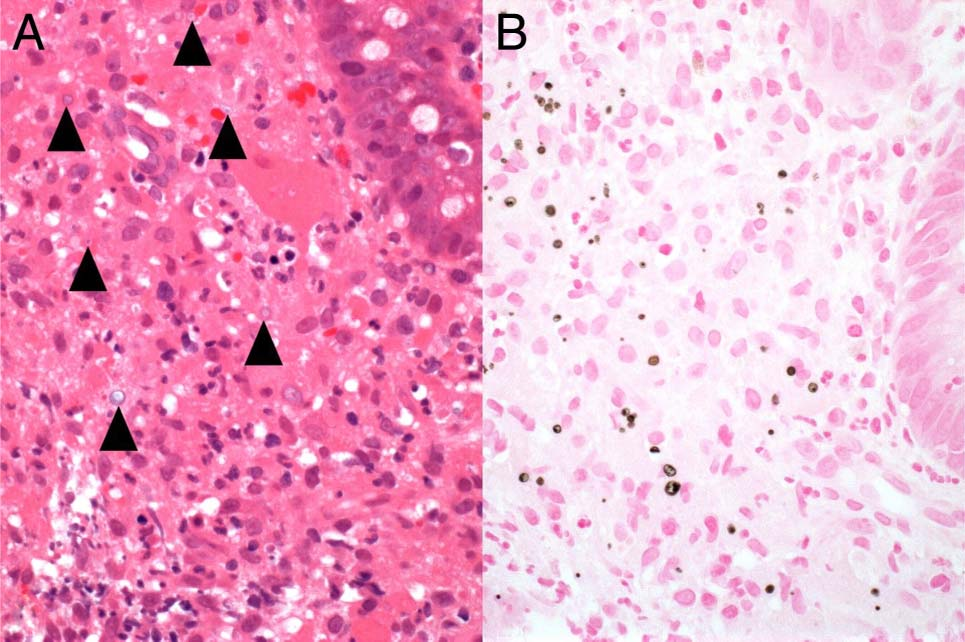
(A) Higher magnification of histiocytes and Michaelis-Gutmann bodies, some displaying a targetoid appearance (arrowheads). (B) Michaelis-Gutmann bodies highlighted by von Kossa stain for calcium phosphate. 40× magnification.

Malakoplakia is a rare granulomatous condition, which results from an atypical inflammatory response to infection, commonly observed in individuals with acquired immunosuppression.^1^ It has been reported in every organ, but preferentially affects the genitourinary tract, with the gastrointestinal tract the second most frequent site (particularly the left colon) and is often associated with Gram-negative bacilli infection. Malakoplakia arises from dysfunctional killing and elimination of bacteria by the macrophage phagolysosome. The diagnosis is made histologically by confirming the presence of von Hansemann cells and Michaelis-Gutmann bodies, which demonstrate the accumulation of enlarged macrophages with calcified cores, containing undigested bacteria and calcium phosphate (Figures 1 and 2).^1^ The natural history of the condition is that, untreated, it results in progressive inflammation, macrophage proliferation, and fibrosis of the affected organ. In the present case, some fibrostenosis was seen in the strictured area in the left colon on the colectomy specimen, but there was no evidence of fibrosis on the mucosal biopsy specimens before colectomy.

Although treatment is not standardized, it usually involves systemic antibiotics, limiting immunosuppression, and, in rare circumstances, surgical excision.^1^ Antibiotics with high intracellular penetrance within macrophages are preferred, although the optimal duration of therapy is unclear. Reduction of immunosuppression is considered a key adjunctive approach to antibiotic therapy. Agents with leukotoxicity such as azathioprine are especially considered to increase the risk of malakoplakia.^2,3^ Surgical excision is required in cases, which are nonresponsive, such as those with progressive end organ fibrosis or development of pseudotumor with a mass effect.^4^

In the present case, the patient had a decade-long history of azathioprine use, alongside over 5 years of biologics, with 3 different agents. Endoscopic examination revealed malakoplakia, and considering the patient's history of a *C. difficile* infection, rifaximin was preferred over ciprofloxacin. However, progressive formation of a stricture, alongside persistent symptoms and endoscopic inflammation, resulted in successful proctocolectomy and permanent end ileostomy.

In conclusion, malakoplakia is an uncommon and rare granulomatous disease that can compromise any organ, with a propensity for the left colon. Michaelis-Gutmann bodies are pathognomonic for diagnosis, but they may be missing in the third stage of the malakoplakia, when there is a progressive fibrous tissue, which may lead to bowel obstruction. It typically affects patients with chronic diseases who are immunosuppressed. We report the first malakoplakia case complicating UC in a patient requiring high doses of combination therapy. Knowledge of this uncommon pathology may lead to better treatment and selection of immunosuppressives in the future, potentially avoiding complications, which require surgical management.

## DISCLOSURES

Author contributions: Study supervision: V. Jairath. Acquisition, analysis, and interpretation of the data: L. Alphonsus, TA De Silva, D. Driman, and V. Jairath. Drafting of the manuscript: L. Alphonsus, TA De Silva, D. Driman, and V. Jairath. Critical revision of the manuscript for important intellectual content: L. Alphonsus, TA De Silva, DD, and V. Jairath.

Financial disclosure: V. Jairath has received has received consulting/advisory board fees from AbbVie, Alimentiv Inc., Arena pharmaceuticals, Asahi Kasei Pharma, Asieris, AstraZeneca, Bristol Myers Squibb, Celltrion, Eli Lilly, Ferring, Flagship Pioneering, Fresenius Kabi, Galapagos, GlaxoSmithKline, Genentech, Gilead, Janssen, Merck, Mylan, Pandion, Pendopharm, Pfizer, Protagonist, Reistone Biopharma, Roche, Sandoz, Second Genome, Takeda, Teva, Topivert, Ventyx, and Vividion and speaker's fees from, AbbVie, Ferring, Galapagos, Janssen Pfizer Shire, Takeda, and Fresenius Kabi. All other authors have no conflicts of interest.

Informed consent was obtained for this case report.
